# Potential of Berry Fruit Pomace for Application in Processed Meat as Natural Alternative to Nitrates and Nitrites

**DOI:** 10.3390/molecules31162788

**Published:** 2026-08-11

**Authors:** Patrycja Skwarek-Jóźwiak, Małgorzata Karwowska

**Affiliations:** Sub-Department of Meat Technology and Food Quality, Department of Animal Food Technology, University of Life Sciences in Lublin, Skromna 8, 20-704 Lublin, Poland; patrycja.skwarek@up.edu.pl

**Keywords:** fruit industry by-products, berry pomace, red currant (*Ribes rubrum*), reduction of nitrogen compounds, antioxidant, antimicrobial activity, clean label, zero waste

## Abstract

In recent years, increasing attention has been paid to limiting the use of nitrates and nitrites in meat products due to concerns regarding their potential health implications. Despite their important technological role in ensuring microbiological safety, oxidative stability, and the development of sensory characteristics of meat products, their use raises health concerns among consumers. These concerns are primarily associated with the potential formation of N-nitrosamines, some of which have been classified possible human carcinogens. In this context, there is growing interest in natural functional additives consistent with the clean-label concept and the zero-waste approach, including raw materials derived from fruit-processing by-products. Fruit-processing by-products, especially berry pomace, are a rich source of phenolic compounds with antioxidant and antimicrobial properties. Current evidence indicates that the bioactive compounds present in berry pomaces effectively reduce lipid oxidation and exhibit antimicrobial potential, which may contribute to inhibiting the growth of undesirable microorganisms in meat products. However, the effectiveness of this activity depends on several factors, including the type of berry pomace, its phenolic profile, the form of application (e.g., powder, extract, or freeze-dried material), the concentration applied, the type of meat product and the storage conditions. Consequently, berry pomaces should be regarded as natural ingredients supporting nitrite reduction strategies in meat products rather than as complete substitutes for nitrites. Furthermore, although berry pomaces exhibit considerable functional potential, their effectiveness in reducing nitrite levels depends on the type of meat product, its formulation, and the applied processing conditions. Among berry pomaces, red currant pomace (*Ribes rubrum*) has recently attracted increasing scientific interest because of its rich phenolic profile and promising functional properties. However, compared with chokeberry, blueberry, blackberry, and blackcurrant pomaces, relatively few studies have investigated its application in meat products. Therefore, further research is needed to confirm its technological functionality, antimicrobial efficacy, and potential contribution to nitrite-reduction strategies under different meat-processing conditions.

## 1. Introduction

Contemporary epidemiological studies suggest that frequent consumption of processed meat products may be associated with an increased risk of certain non-communicable diseases, including cardiovascular diseases, type 2 diabetes, and colorectal cancer. In response to growing consumer health awareness and increasing interest in healthy dietary patterns, substantial research efforts have been directed towards improving the composition of processed meat products, with particular emphasis on enhancing their nutritional value, ensuring their safety, and reducing their potential adverse effects on human health [1].

In this context, nitrates and nitrites, commonly used in meat processing are attracting considerable attention. Added to meat products, they perform important technological functions, inhibiting the growth of pathogenic microorganisms, including *Clostridium botulinum* and slowing down lipid oxidation. In addition, nitrite is converted into nitric oxide (NO), which reacts with myoglobin to form nitrosylmyoglobin. During heat treatment, this pigment is converted into nitrosylhemochrome, responsible for the characteristic stable pink–red colour of cured meat products. These reactions play a crucial role in the development and stabilization of the typical cured meat appearance. These compounds also contribute to the development of characteristic sensory properties of meat products. At the same time, by-products of nitrogenous compound metabolism, especially nitrosamines, raise health concerns due to their potential carcinogenic effects, justifying strict control and regulation of their use in the meat industry [2,3,4]. Therefore, the development of functional meat products is currently focused on reducing the content of these synthetic preservatives or partially replacing them with alternative technological solutions, provided that microbiological safety is not compromised, particularly with respect to the inhibition of *Clostridium botulinum*, while maintaining oxidative stability and the desired sensory characteristics of the products [5,6].

These initiatives align with the trend toward developing innovative meat products with reduced nitrogen content, meeting both market expectations and consumer health needs. This phenomenon is closely linked to the dynamic development of the “clean label” concept, which involves reducing synthetic additives in favor of natural ingredients that are more acceptable to consumers [7]. At the same time, these ingredients should maintain key technological properties, including oxidative stability, microbiological safety, and the sensory quality of meat products. Consumer acceptance of plant-derived by-products depends not only on their natural origin but also on their ability to preserve the quality and shelf life of the final product.

In recent years, there has also been a growing interest in using fruit processing by-products as natural additives to meat products. This trend stems from both the need to reduce the use of synthetic preservatives and the search for solutions enabling more efficient use of raw materials generated during processing [8]. Fruit industry by-products, especially berry pomace, are a rich source of phenolic compounds with well-documented antioxidant and antimicrobial properties. As a result, they may represent a potential alternative to synthetic preservatives, helping to reduce lipid oxidation and the development of undesirable microflora in meat products [9]. Particular attention has been paid to red currant pomace, which, due to its rich phenolic profile, demonstrates high functional potential. Their use may be a promising approach for developing meat products with reduced synthetic preservative content, while increasing oxidative stability and the potential health-promoting value of the final products [10]. To date, research has primarily focused on chokeberry, blackcurrant, blueberry, and blackberry pomaces, whose functional properties have been extensively documented. In contrast, relatively few studies have investigated red currant pomace (*Ribes rubrum*), despite its rich content of phenolic acids, flavonoids, anthocyanins, and vitamin C with well-documented antioxidant and antimicrobial properties. This limited evidence indicates that red currant pomace is a promising, yet insufficiently explored, natural ingredient for supporting nitrite-reduction strategies while maintaining the technological quality, oxidative stability, and microbiological safety of meat products.

The aim of this review is to present the current state of knowledge on the use of fruit processing by-products, as alternative additives to meat products, with particular emphasis on the potential of berry pomace in reducing the use of synthetic preservatives. This approach supports the development of more sustainable and health-promoting dietary practices.

A systematic literature search was conducted to identify studies addressing the antioxidant and antimicrobial potential of berry fruit pomace in meat products with reduced levels of nitrogen compounds. The literature search was performed using the Web of Science Core Collection database. The search strategy combined the following keywords: (“fruit industry by-products” OR “fruit by-products” OR “berry pomace” OR “red currant pomace”) AND “meat products” AND (“nitrite reduction” OR “nitrate reduction” OR “reduced nitrite” OR “reduced nitrate”) AND “zero waste”. Only original research articles and review papers published in peer-reviewed journals and written in English were considered. The initial search identified more than 130 records (in the time period 15 April 15–28 April 2026). After removing duplicates and screening titles and abstracts for relevance, full-text articles were assessed according to the predefined inclusion and exclusion criteria. Studies that did not address the scope of the review or lacked sufficient experimental relevance were excluded. Ultimately, 95 articles were included in the review. In addition, the reference lists of the selected publications were manually screened to identify further relevant studies not retrieved during the database search.

## 2. Berry Fruit Pomace as a Source of Bioactive Compounds with Technological Potential

Berries are among the most important fruits in Europe, both in terms of production volume and economic importance. According to Eurostat, in 2023, berry production in the European Union amounted to approximately 700,000 tons, whereas the forecast of approximately 1,020,000 tons by 2028. The most commonly consumed species include blueberries (*Vaccinium corymbosum*), raspberries (*Rubus idaeus*), blackberries (*Rubus fruticosus*), black chokeberries (*Aronia melanocarpa*), strawberries (*Fragaria ananassa*), white currants (*Ribes niveum*), black currants (*Ribes nigrum*) and red currants (*Ribes rubrum*) [11,12]. They are valued not only for their sensory qualities but also for their high content of bioactive compounds, including polyphenols, anthocyanins, flavonoids, and phenolic acids, which determine their health-promoting properties and potential use in functional foods.

During fruit processing, particularly in the production of juices, jams, and concentrates, pomace is a by-product consisting of peels, seeds, and pulp residues. It is estimated that pomace constitutes approximately 20–25% of the processed fruit weight. However, this proportion varies considerably depending on the fruit species, cultivar, maturity stage, and the processing technology applied, particularly the juice extraction method and processing efficiency [13]. Pomace contains a significant amount of polyphenols and antioxidants, making it a valuable raw material for the isolation of bioactive compounds with technological potential. It also contains dietary fiber, pectins, proteins, lipids, vitamins and numerous phenolic compounds and anthocyanins [14,15,16].

Black currant (*Ribes nigrum*) is a particularly valuable raw material. Fresh black currant fruits are characterized by high content of anthocyanins, including delphinidin-3-O-glucoside, delphinidin-3-O-rutinoside, cyanidin-3-O-rutinoside, and the derivatives malvidin, petunidin, and peonidin, as well as flavonols such as kaempferol and quercetin, and phenolic acids, including chlorogenic acid and caffeic acid-O-glucoside [17,18]. Fresh fruits also contain vitamin C, total sugars (5–8%), minerals (0.60–0.96%), dietary fiber (5%), and pectin (1%) [19]. During juice processing, a substantial proportion of polyphenols associated with the fruit skins and seeds, together with dietary fiber and pectins, remains in the pomace, although their content depends on the fruit cultivar and the processing technology applied. Consequently, black currant pomace remains a valuable source of bioactive compounds for food applications. Black currant has a wide range of health-promoting properties, including antioxidant, anti-inflammatory, immune-supporting and metabolism-regulating effects [20].

Red currants (*Ribes rubrum*) are characterized by high vitamin C and anthocyanin content, primarily cyanidin-3-O-rutinoside and cyanidin-3-O-(2″-O-xylosyl)rutinoside. These fruits also contain flavonoids such as rutin and quercetin, as well as phenolic acids, including caffeic acid and 4-hydroxybenzoic acid-O-hexoside [13,21,22,23]. Owing to their rich composition of bioactive compounds, red currants exhibit strong antioxidant and anti-inflammatory properties and are considered a promising component of a health-promoting diet. However, further clinical studies are needed to confirm their role in the prevention of chronic diseases [24,25].

Black chokeberry (*Aronia melanocarpa*) is a fruit with exceptionally high anthocyanin and proanthocyanidin content, reaching approximately 1500 mg/100 g and 5000 mg/100 g dry weight (DW) [26,27]. It also contains flavonols, quercetin glycosides, and hydroxycinnamic acids, such as chlorogenic and neochlorogenic acids. On a fresh weight (FW) basis, dietary fiber constitutes approximately 5.6% of the fruit, with a predominance of cellulose, hemicellulose, and pectins [16,28]. Technological processes, including juice pressing, generate significant amounts of pomace, which can constitute up to 16% of the raw material and contain substantial levels of polyphenols and antioxidants [29].

Raspberries (*Rubus idaeus*) contain various anthocyanins, including cyanidin-3-O-sophoroside, cyanidin-3-O-(2″-O-glucosyl)rutinoside, cyanidin-3-O-sambubioside, cyanidin-3-O-glucoside, as well as traces of ellagic acid and quercetin derivatives [30]. Blueberries, in turn, are characterized by the presence of delphinidin-3-O-galactoside, cyanidin-3-O-galactoside, petunidin-3-O-galactoside and malvidin-3-O-galactoside, as well as 5-O-feruloylquinic acid and trace amounts of quercetin glycosides [17,30]. These fruits also contain numerous phenolic acids, such as gallic, chlorogenic, ellagic and ferulic acids.

Cranberries (*Vaccinium* spp.) are a source of vitamin C, anthocyanins, and phenolic acids, including p-coumaric, caffeic, and ferulic acids, which determine their antioxidant activity [17]. Anthocyanins constitute the second most important group of antioxidant compounds in cranberries, and their presence in pomace highlights its role as a valuable source of antioxidants [31].

The chemical composition of berry pomace varies depending on the fruit species, but the dominant component is dietary fiber. Chokeberry pomace powders contain approximately 3.6% fat, 6.0% protein, 28.8% carbohydrates and 57.8–59.5% dietary fiber on a dry matter (DW) [32]. Raspberry pomace is characterized by 9.1–11.1% fat, 8.7–10.0% protein, and 54.2–59.5% fiber; strawberry pomace contains 3.4% fat, 9.2% protein, and 33.9% fiber and black currant pomace contains 20.2% fat, 15.7% protein and 59.1% fiber (DW) with a predominance of the insoluble fraction [32,33]. Red currant pomace contains approximately 14.2% fat, 11.8% protein, 12.7% carbohydrates, and 58.1% fiber (DW), mainly insoluble. The presence of seeds in black currant and red currant pomace powder additionally increases the content of bioactive compounds, including polyunsaturated fatty acids, phytosterols and tocopherols.

The literature indicates that fruit pomace is a promising raw material for functional food applications owing to its high content of bioactive compounds with antioxidant and antimicrobial properties. These properties may support the oxidative and microbiological stability of meat products, highlighting their potential as natural functional ingredients. This is particularly relevant in fermented meat products, where fruit pomaces may help maintain the technological quality and microbiological safety of products with reduced nitrate and nitrite levels [30].

Berry fruit pomaces represent a promising source of bioactive compounds (Table 1) with potential applications in meat products, particularly as natural functional ingredients supporting nitrite-reduction strategies. However, despite the growing body of research on berry pomaces, evidence regarding the application of red currant pomace in meat products remains limited. Therefore, further studies are needed to confirm its technological functionality, antimicrobial efficacy, and potential role in reducing nitrate and nitrite levels while maintaining product quality and safety.

## 3. Potential Mechanisms of Activity of Berry Fruit Pomace in Meat Products with Reduced Nitrogen Compound Content

### 3.1. Nitrates and Nitrites in Processed Meat

Nitrates and nitrites are typical food preservatives used in meat processing, both as a sodium and potassium salt. The use of nitrates and nitrites in meat products offers both technological and food safety benefits, as they inhibit the growth of pathogenic microorganisms (especially *Clostridium botulinum*), delay lipid oxidation (through the oxygen deletion, nitric oxide can react with radicals interrupting radical chain reactions and bind to transitional metals), and contribute to the development of characteristic flavor (several compounds are formed when nitrite is bound to lipids and proteins) and color (via interaction with the myoglobin in muscle). Antimicrobial effect of nitrite is related to inhibiting metabolic enzymes of bacteria, limiting oxygen uptake, and breaking the proton gradient [2]. At the same time, the literature indicates the risk of nitrosamine formation in the reaction of nitric oxide with secondary amines and several N-nitrosamines have been classified as possible human carcinogens [34]. For this reason, the use of nitrates and nitrites in meat products is strictly regulated in the European Union, with maximum permitted levels established by Commission Regulation (EU) 2023/2108. Commission Regulation (EU) 2023/2108 amends previous EU legislation to significantly reduce the maximum permitted limits of nitrites and nitrates used as food additives. These reductions aim to minimize consumer exposure to potentially carcinogenic nitrosamines while maintaining food microbiological safety. Additionally, several studies have investigated natural alternatives to nitrite in meat products. Wang et al. [35] demonstrated that rose extract could effectively reduce the need for nitrite in semi-dried fermented sausages. Sausages formulated with rose extract exhibited superior quality compared with those containing 150 mg/kg sodium nitrite, with the most favorable results obtained using a combination of 10% rose extract and 80 mg/kg sodium nitrite. In another study, Ozaki et al. [36] incorporated radish powder (0.5% or 1.0%) together with oregano essential oil (100 mg/kg) into fermented cooked pork and beef sausages. This formulation enhanced product color and reduced the growth of mesophilic microorganisms. However, it was ineffective in preventing lipid oxidation. Zhu et al. [37] presented research in which they investigated lowering NaNO_2_ to a level of 50 mg/kg. They demonstrated that the incorporation of *Lactobacillus plantarum* could partially substitute for nitrite in pork sausages. Their results showed that the combination of 50 mg/kg NaNO_2_ and 7 log CFU/g of *L. plantarum* achieved effects comparable to those obtained with 100 mg/kg NaNO_2_ alone.

### 3.2. Benefits of Using Berry Fruit Pomace in Meat Products

Due to the potential toxicity of nitrates and nitrites, their use is regulated, and one of the key directions in meat technology development is the search for solutions that improve the nutritional value and safety of products while reducing the use of synthetic additives [3,4]. In this context, berry pomace aligns with the principles of the circular economy, enabling the valorization of fruit industry by-products and their use as valuable functional additives [38].

Berry fruit pomace, a by-product of the fruit processing industry, is a raw material with high potential for application in food technology, particularly in meat processing. Interest in its use stems from both the need to enhance shelf life and microbiological safety of meat products and the growing consumer demand for “clean label” products with simplified compositions and reduced synthetic additives [39,40]. In meat products with reduced nitrate and nitrite levels, berry pomaces may support oxidative stability and help inhibit the growth of undesirable microorganisms owing to their antioxidant and antimicrobial properties. Their application may therefore constitute an important element of nitrate and nitrite reduction strategies; however, they cannot replace all the technological functions performed by these curing agents in meat products. Research conducted by Olanwanit and Phahom [41] demonstrated that Mao berry (*Antidesma bunius*) extracts, particularly from the peel, are highly effective alternatives to sodium nitrite in emulsion sausages. Added at concentrations of 1.25% to 2.50%, they successfully delayed lipid oxidation and inhibit microbial growth over a 30-day refrigerated storage period, yielding results comparable to traditional nitrite preservatives.

One of the most important mechanisms of action of berry fruit pomace in meat products is its ability to limit oxidative processes. Meat and meat products, especially those rich in unsaturated fatty acids, are susceptible to lipid peroxidation, which leads to the formation of reactive oxidation products. This process leads to a deterioration of sensory properties such as flavor, aroma and color, as well as to a reduction in nutritional value and shelf life [42]. The most common group of bioactive compounds in berry pomace are phenolic compounds, including phenolic acids, flavonoids, anthocyanins, and tannins, which are characterized by the ability to scavenge free radicals and reduce oxidative stress [31]. Their mechanism of action includes neutralizing free radicals, chelating metal ions that initiate oxidation reactions, inhibiting lipid peroxidation, and reducing the formation of peroxides and hydroperoxides, which is considered crucial for protecting cellular structures from oxidative stress [43]. The high polyphenol content in pomace results from its concentration in the fruit skin and seed. It is estimated that approximately 28–35% of phenolic compounds are found in the peels and 60–70% in the skin [44]. Anthocyanins play a particularly important role in antioxidant activity, and their ability to neutralize reactive oxygen species strongly depends on their chemical structure [45]. Therefore, the addition of berry pomace to meat products can effectively reduce oxidation and improve oxidative stability, thereby reducing the need for synthetic preservatives in meat processing.

Oxidative deterioration also affects muscle proteins, leading to the formation of protein carbonyls, oxidation of sulfhydryl groups and protein cross linking. These modifications impair the functional properties of proteins by reducing their water-holding capacity, solubility, emulsifying properties, and gel forming ability, ultimately negatively affecting the texture, juiciness and processing yield of meat products. Moreover, lipid and protein oxidation are closely interconnected processes. Secondary lipid oxidation products, such as malondialdehyde (MDA), 4-hydroxynonenal (4-HNE) and other reactive aldehydes, may further promote protein oxidation, whereas oxidized proteins can accelerate subsequent lipid deterioration, creating a self-propagating cycle of oxidative reactions [42]. Furthermore, polyphenols present in berry pomaces may influence the formation of volatile compounds responsible for the flavour and aroma of meat products. Numerous studies have demonstrated that reducing lipid oxidation also decreases the formation of aldehydes, particularly hexanal, which is widely recognized as a marker of lipid oxidation. However, the technological effectiveness of berry pomace largely depends on its inclusion level. While moderate concentrations (0.5–2.5%) generally improve oxidative stability without compromising product quality [46,47] excessive additions may adversely affect the physicochemical and sensory properties of meat products. For example, the incorporation of 8% freeze-dried apple pomace into turkey sausages resulted in reduced lightness, redness, hardness, springiness, cohesiveness, and overall sensory acceptability, despite improving antioxidant activity [48]. Similarly, the addition of 9% dried apple pomace to chicken sausages increased hardness and negatively affected texture [49], whereas 15% apple pomace incorporated into mutton nuggets significantly reduced flavour, texture and overall acceptability [50]. These findings indicate that excessive levels of fruit pomaces may increase bitterness, astringency, and plant-derived flavour notes while negatively affecting colour, texture, and consumer acceptance, emphasizing the need to optimize the inclusion level for each meat product individually.

In addition to their antioxidant properties, polyphenols present in berry fruits also exhibit antimicrobial activity, further highlighting their biological potential [51,52,53,54,55]. The antimicrobial effects of berry pomace have been demonstrated in meat products, where they inhibited the growth of pathogens such as *Listeria monocytogenes*, *Campylobacter jejuni* and *Escherichia coli* [21,54,55]. However, considering the key role of nitrates and nitrites in controlling *Clostridium botulinum* in cured meat products, further studies are needed to determine whether berry pomaces can also contribute to maintaining protection against this pathogen when nitrate and nitrite levels are reduced. Bioactive compounds present in berry pomace, particularly polyphenols and flavonoids, can interact with microbial cellular structures. Their mechanism involves destabilization of the cytoplasmic membrane and disruption of bacterial cell structure. Hydrophobic compounds can penetrate the bacterial lipopolysaccharide layer and damage cell membranes [56]. Polyphenols may also inhibit the activity of extracellular enzymes of microorganisms and limit the availability of nutrients necessary for their growth and proliferation [57]. Microbial growth inhibition may also be influenced by the low pH of pomace, which creates unfavorable conditions for many microorganisms [55]. An important aspect of this activity is the presence of naturally occurring organic acids in berry pomaces, whose antimicrobial effectiveness depends on pH. In an acidic environment, these acids remain undissociated, facilitating their penetration through bacterial cell walls. Once inside the cell, where the cytoplasmic pH is relatively neutral, they dissociate into protons and anions, lowering the microorganism’s internal pH. Attempts to compensate for these changes slow bacterial growth and, under unfavorable conditions, can lead to cell death [58]. Furthermore, pomace contains tannins, which can enhance antimicrobial activity by damaging cell membranes and inhibiting enzymes.

The color of meat products is a key factor influencing food quality and consumer acceptance. Color deterioration is associated with oxidative processes, including lipid oxidation and myoglobin degradation, which leads to reduced shelf life and lower consumer acceptance [42]. In this context, berry pomace can act as a natural colorant in meat products. The main groups of pigments present in berry raw materials include anthocyanins, carotenoids, and betalains, although their proportion varies by fruit species [59]. These compounds can act as natural colorants in meat products, enabling attractive color without the need for synthetic additives [60]. However, it should be emphasized that, unlike nitrosylmyoglobin formed during the curing process, anthocyanins exhibit lower colour stability, and their stability is strongly influenced by pH, temperature, oxygen availability and light exposure. Consequently, the addition of berry pomace may alter the colour characteristics of meat products, resulting in a darker appearance or the development of red, purple, or violet hues, depending on the type and concentration of pomace used as well as the processing conditions.

Berry pomace is also a valuable source of dietary fiber, both soluble and insoluble. The soluble fiber content in berry pomace powders can range from 4.92 to 12.74 g/100 g dry matter [61]. Soluble fractions, primarily pectins, exhibit gelling, thickening, and stabilizing properties. As a result, they can increase the water-holding capacity of meat products and improve the stability of fat-water emulsions. These mechanisms improve the juiciness and texture of meat products. Insoluble fiber fractions, such as cellulose, hemicellulose, and lignin, contribute to increasing the volume and structural integrity of the products. Consequently, they may improve consistency, firmness, and overall texture-related sensory perception [62,63]. Furthermore, the ability of dietary fiber to retain water and stabilize emulsions may improve cooking yield and contribute to consumer acceptance by enhancing the texture and overall sensory quality of meat products.

Berry pomace is also a source of oils rich in polyunsaturated fatty acids (PUFA), including linoleic acid (omega-6) and α-linolenic acid (omega-3). In addition to PUFA, oils derived from this raw material are rich in phytosterols, mainly β-sitosterol, as well as campesterol, stigmasterol, and stigmastanol, with total contents ranging from 101.6 to 168.2 mg/100 g. Tocopherols (α, β, γ, δ) with antioxidant activity are also present [64]. As a result, these components may improve the lipid profile of meat products and act as natural emulsifiers. Although these compounds are not directly involved in reducing nitrate and nitrite levels, they may support the oxidative stability of meat products and act synergistically with other bioactive compounds present in berry pomace, thereby contributing to the maintenance of product quality and nutritional value [65,66].

Berry pomace is also a valuable source of vitamins and minerals, including iron, calcium, magnesium, potassium, vitamin C, vitamin K, and selected B vitamins such as folic acid [67]. These components play important physiological roles in the human body. Vitamin C exhibits strong antioxidant properties and enhances the bioavailability of non-heme iron. Magnesium and calcium are essential for the proper functioning of the skeletal and muscular systems, while potassium helps regulate blood pressure and electrolyte balance [68,69]. Additionally, several studies indicate that regular consumption of polyphenols and anthocyanins present in berry fruits may be associated with a reduced risk of chronic diseases, including cardiovascular diseases, type 2 diabetes and certain cancers [70,71,72]. Therefore, the incorporation of berry pomace into meat products may increase the content of selected bioactive compounds and micronutrients, potentially improving their nutritional profile.

Protein–polyphenol interactions play an important role in determining the quality of meat products containing berry pomace. Polyphenols, including flavonoids, anthocyanins, and tannins, can interact with meat proteins through both non-covalent interactions (mainly hydrogen bonds and hydrophobic interactions) and covalent bonds formed following the oxidation of phenolic compounds [73,74,75,76]. These interactions influence the structural and functional properties of meat proteins, including water-holding capacity, gel formation, emulsion stability and texture. They may also affect colour through interactions with myoglobin, influence protein digestibility, and reduce oxidative changes in proteins and lipids, thereby contributing to the maintenance of antioxidant activity and the overall quality of meat products during storage [77,78].

Myofibrillar proteins, which constitute approximately 50–55% of muscle proteins, play a key role in shaping the functional properties of meat, such as water binding, gelation and emulsification [79]. However, during meat processing, transport and storage, they are oxidized by reactive oxygen species, leading to a deterioration in the quality and nutritional value of meat products [80]. Iron ions play an important role in these processes by initiating the Fenton reaction, which generates highly reactive hydroxyl radicals that degrade lipids and proteins [81]. Phenolic compounds can limit these unfavorable processes by neutralizing free radicals and chelating metal ions, including iron, which limits their participation in oxidation reactions [82]. In the Fenton system, they exhibit both antioxidant and prooxidant effects, depending primarily on their concentration and matrix properties [83]. Low concentrations of polyphenols inhibit the formation of carbonyl groups, limit the loss of sulfhydryl groups and stabilize the structure of myofibrillar proteins, improving their gelling properties and the texture of meat products. However, high concentrations can lead to prooxidant effects, causing denaturation, aggregation and deterioration of protein textural properties [84]. Protein–polyphenol interactions also have biological significance. These complexes can increase the stability and bioavailability of polyphenols in the gastrointestinal tract, protecting them from enzymatic degradation and enabling their transport to the large intestine, where they exert antioxidant effects and regulate the intestinal microbiota. Their potential role in reducing oxidative stress and regulating gut microbiota has also been suggested [85]. The impact of these interactions on food sensory attributes, such as color, flavor, and texture, is also significant, particularly when using polyphenol-rich meat and plant-based additives. These phenomena create opportunities to develop innovative meat products with enhanced health-promoting value [86]. These mechanisms have also been confirmed in studies conducted on meat products enriched with berry pomace. Cegiełka et al. [86,87] demonstrated that the incorporation of chokeberry pomace into pork burgers improved oxidative stability and limited oxidative changes during refrigerated storage. Enriching foods with phenolic compounds can enhance their health-promoting properties, but interactions between these compounds and food components, especially proteins, can affect both antioxidant activity and protein digestibility, underscoring the need for further research in this area.

## 4. Methods of Preparation and Use of Berry Pomace in Meat Products

Depending on the processing technology used, pomace can be used in various forms in meat processing, including extracts, powders obtained by drying or freeze-drying, fermented products and microencapsulated ingredients. The use of these forms enhances the stability of bioactive compounds and their effective incorporation into the meat product matrix. The use of various forms of berry fruit pomace in meat products are presented in Table 2. One of the most common uses of berry pomace in food technology is the production of extracts rich in bioactive compounds. The extraction process plays a key role in isolating functional compounds from fruits and their by-products. Traditional extraction methods, such as solvent extraction, are widely used due to their simplicity and relatively high yields; however, they have limitations, including long processing times, the need for large volumes of solvent, and the risk of thermal degradation of sensitive bioactive compounds [88]. In recent years, modern extraction techniques have been increasingly used, enabling higher process efficiency and better preservation of the biological properties of isolated compounds. The most commonly used methods include ultrasound-assisted extraction, microwave-assisted extraction, pressurized liquid extraction, high-pressure extraction, and supercritical fluid extraction [89,90]. These methods enable the effective recovery of bioactive compounds while reducing solvent consumption and minimizing environmental impact.

Depending on the intended application, berry pomace extracts and powders exhibit distinct technological characteristics that determine their suitability for different meat products. Due to their high concentration of bioactive compounds, extracts are primarily used as sources of natural antioxidants and antimicrobial compounds while having a relatively limited impact on the structural and sensory characteristics of meat products. In contrast, dried or freeze-dried berry pomace powders retain the entire plant matrix, including polyphenols, dietary fiber and other bioactive constituents, thereby influencing functional and technological properties such as water-holding capacity, emulsion stability, and texture. However, increasing the level of pomace powder incorporation may also affect the colour and sensory characteristics of the final product. Therefore, the selection of the most appropriate form of berry pomace should be based on the intended technological function and the characteristics of the specific meat product.

Berry fruit extracts have found numerous applications in meat products as natural antioxidants and antimicrobials. Muzolf-Panek et al. [99] showed that the incorporation of 2% blueberry preparation into pork meatloaf stored for 12 days at 8 °C increased the oxidative stability of the product. The authors evaluated TBARS values, antioxidant activity and colour stability, confirming the protective effect of the berry additive. Similar results were obtained in studies on blackberry extracts. Ganhão et al. [91] demonstrated that the addition of 3% blackberry extract to pork burger patties stored for 12 days at 2 °C significantly reduced the formation of protein oxidation products, while colour and texture analyses confirmed a slower deterioration of product quality during storage. In a subsequent study, the same authors also confirmed that the addition of 3% blackberry extract effectively reduced lipid oxidation, as evidenced by significantly lower TBARS values throughout refrigerated storage [92]. Black currant extracts also exhibit strong antioxidant activity in meat products. Jia et al. [93] demonstrated that the addition of 5, 10 and 20 g/kg of black currant extract to pork patties stored for 9 days at 4 °C significantly reduced TBARS values and limited the formation of protein carbonyls. The study also evaluated sulfhydryl group content and colour stability, confirming effective inhibition of both lipid and protein oxidation processes. Extracts can also be obtained from berry pomace. Babaoğlu et al. [21] analyzed the effect of aqueous extracts from chokeberry, blackberry, red currant, and blueberry pomace on the oxidative stability of beef cutlets stored under refrigerated conditions. All variants containing the extracts showed significantly lower TBARS values than the control sample, with the highest antioxidant activity observed in the blackberry pomace extract. Furthermore, the study demonstrated inhibition of microbial growth, including mesophilic aerobic bacteria, psychrotrophic aerobic bacteria and *Escherichia coli*. This effect is attributed to the presence of phenolic compounds and ascorbic acid, which neutralize reactive oxygen species and inhibit oxidation processes in products [94,100]. Raspberry pomace extracts also exhibited beneficial effects in meat products. Kryževičūtė et al. [101] evaluated pressurized ethanol/water extracts (0.3 and 0.5%) obtained from raspberry pomace in beef burgers stored at 4 °C under modified atmosphere packaging. The extracts significantly inhibited lipid oxidation, reduced the growth of *Enterobacteriaceae* and *Pseudomonas* spp., and maintained the sensory quality of burgers during storage.

Drying is a fundamental process for stabilizing fruit pomace and extending its shelf life. Removing water limits microbial growth and slows down enzymatic and non-enzymatic reactions [95]. The most commonly used method in the food industry is hot air convection drying. This process involves simultaneous heat and mass transfer between the drying medium and the product surface, although prolonged exposure to high temperatures can degrade bioactive compounds, especially polyphenols and anthocyanins [102].

Several studies have demonstrated that drying temperatures exceeding approximately 70 °C promote considerable degradation of thermolabile compounds, resulting in significant reductions in total phenolic content, anthocyanin concentration and antioxidant activity. Conversely, drying performed at moderate temperatures (approximately 50–60 °C) provides a more favourable balance between the preservation of bioactive compounds and processing efficiency while substantially reducing production costs [103].

An alternative to conventional drying methods is freeze-drying (lyophilization), which involves removing water from previously frozen material by sublimating ice under reduced pressure. This method preserves the structure of the raw material and minimizes thermal degradation of bioactive compounds [22]. Despite its high energy costs, freeze-drying is widely used in the production of functional foods, where maintaining the high biological quality of the raw material is crucial [104]. Freeze-drying is widely recognized as the most effective preservation method for berry pomace because the low processing temperature minimizes thermal degradation, ensuring superior retention of polyphenols, anthocyanins, vitamins and antioxidant activity. Consequently, freeze-dried berry pomaces generally contain higher concentrations of bioactive compounds than materials dried using conventional methods; however, the effectiveness of drying technologies varies depending on berry species and their chemical composition. For example, chokeberry and blueberry pomaces generally exhibit greater phenolic stability during drying than raspberry or strawberry pomaces, whereas blackcurrant pomace is more susceptible to high temperatures because of its high content of thermolabile ascorbic acid. In addition to berry species, the concentration and profile of bioactive compounds are also influenced by cultivar, geographical origin, climatic conditions, fruit maturity, juice extraction technology, and storage conditions prior to processing [105].

Dried berry pomace can be used as a functional additive in meat products. For example, the use of berry pomace in pork patties improved oxidative stability and reduced the formation of volatile lipid oxidation products such as hexanal, 2-heptanal, and octanal [106]. Furthermore, the presence of pomace increased cooking efficiency, attributed to the dietary fiber’s ability to retain water and fat in the meat matrix. Similar effects were observed with chokeberry pomace. Its addition to pork burgers decreased pH and significantly altered color parameters, including a decrease in lightness (L*) and an increase in redness (a*) [86]. These changes were attributed to the presence of anthocyanins, which give the pomace its characteristic dark purple color. Chokeberry pomace also affected the product’s textural properties, increasing hardness with higher inclusion levels [87]. However, despite their numerous technological advantages, the use of dried berry pomace in meat products is also associated with certain limitations. Increasing the level of pomace incorporation may intensify bitterness and astringency due to the high content of polyphenols and tannins. Moreover, the natural pigments present in berry pomace may cause product darkening or the development of purple and reddish hues, while the high dietary fibre content may increase hardness and adversely affect texture and overall sensory acceptance, particularly at higher inclusion levels.

One modern method for increasing the stability of bioactive compounds derived from berry pomace is microencapsulation. This technique involves encapsulating the active ingredient in a carefully selected coating material, which creates a capsule or membrane structure. The resulting system protects the bioactive compounds from environmental factors and enables their controlled release under specific conditions [96,97]. In addition to improving oxidative and thermal stability, microencapsulation protects sensitive compounds against oxygen, moisture, light, and other environmental factors, thereby extending the shelf life of berry-derived functional ingredients. Furthermore, encapsulation can effectively mask undesirable flavours, colours, and astringency associated with certain berry pomaces, improving consumer acceptability while enabling controlled release of bioactive compounds within the food matrix or during gastrointestinal digestion.

Encapsulation processes most often utilize GRAS-recognized materials such as polysaccharides, proteins, and lipids. Typical carriers include maltodextrins, gum arabic, pectins, alginates, whey proteins and gelatin [107]. Depending on the physicochemical properties of the active substance, different encapsulation methods are used, including mechanical techniques (spray drying, freeze-drying), physicochemical methods (such as ionic gelation) and chemical methods [108].

An example of microencapsulation applied to berry pomace is the encapsulation of phenolic compounds derived from blackberry pomace. Studies have shown that spray-drying microencapsulation effectively protects anthocyanins and other phenolic compounds from degradation while maintaining color stability and high antioxidant activity [109]. Similarly, the use of raspberry powder obtained by spray drying improved the quality of vacuum-packaged ground beef. The addition of this ingredient reduced microbial counts and extended the shelf life compared to the control sample [110]. In meat products with reduced nitrite content, microencapsulation may further enhance the effectiveness of berry derived bioactive compounds by protecting them from degradation during processing and storage and enabling their controlled release throughout the product shelf life. Consequently, microencapsulated berry ingredients may help maintain antioxidant and antimicrobial activity, thereby partially supporting the technological functions of reduced nitrite levels, including improved oxidative stability and extended shelf life. Despite these promising results, the industrial implementation of microencapsulation remains challenging. The incorporation of additional encapsulation steps increases production complexity, processing time and manufacturing costs. Therefore, further research should focus on developing economically feasible encapsulation technologies suitable for large scale food production while maintaining high encapsulation efficiency and product stability.

Fermentation is another processing method for fruit industry by-products, enabling improved sensory properties and increased bioactive potential. This process is carried out using microorganisms, primarily lactic acid bacteria and yeasts, which modify the chemical profile of the raw material and influence its flavor, aroma and antioxidant activity [98,111]. During fermentation, microbial enzymes partially degrade plant cell wall poly-saccharides, facilitating the release and increasing the bioaccessibility of phenolic com-pounds, flavonoids and other bioactive constituents that were previously bound within the plant matrix. Consequently, fermentation may significantly enhance the functional properties of berry pomace.

Studies on the use of fermented berries in meat products have shown that the addition of fermented berries to emulsion sausages significantly reduced lipid oxidation processes. Samples containing fermented berries showed TBARS values below 1 mg MDA/kg of product, while control samples reached values up to 2.73 mg MDA/kg during storage [46]. Simultaneously, a reduction in protein carbonyl formation was observed, indicating that fermented plant materials protect the oxidative stability of meat products.

Besides improving antioxidant properties, fermentation may also increase the microbiological stability of berry pomace through the production of organic acids, bacteriocins, and other antimicrobial metabolites. Moreover, fermentation modifies the sensory profile of berry pomace by reducing bitterness and astringency while generating desirable aroma compounds, thereby facilitating its incorporation into meat products without adversely affecting consumer acceptance. Compared with encapsulation technologies, fermentation generally requires lower capital investment and can be more readily implemented under industrial conditions. The process is relatively simple and environmentally friendly. However, its effectiveness depends strongly on microbial strain selection, substrate composition, fermentation time, temperature, pH, oxygen availability and other process parameters. Consequently, achieving consistent product quality remains challenging, and further studies are required to standardize fermentation protocols for different berry pomaces and intended food applications [98,111].

Various processing methods for berry pomace enable its effective use in the meat industry as natural functional ingredients. Berry extracts appear to be particularly suitable for meat products with reduced nitrite content, where their high antioxidant and antimicrobial activity can help maintain oxidative stability and extend shelf life without markedly affecting product texture or sensory characteristics. In contrast, dried and freeze-dried pomace powders are especially promising for comminuted and emulsified meat products, as they contribute not only bioactive compounds but also dietary fibre, thereby improving water holding capacity, emulsion stability and texture. Microencapsulation represents a promising strategy for protecting sensitive bioactive compounds and ensuring their controlled release during processing and storage, whereas fermentation may further enhance antimicrobial activity, facilitate product acidification and improve flavour characteristics. Therefore, the selection of the appropriate form of berry pomace should be based on the technological requirements and quality attributes of the specific meat product.

## 5. Conclusions

This review highlights the potential of berry pomace as an innovative ingredient in the creation of functional meat products. The addition of berry pomace adds valuable antioxidants, dietary fiber, natural colorants, oils, vitamins, and minerals to these products, enhancing their nutritional value and influencing microbiological characteristics. The use of berry pomace in processed meats also supports sustainable dietary practices by minimizing waste and fostering a circular economy. However, this review identifies several challenges, including the variability of pomace composition and the introduction of undesirable flavors. Regarding the use of berry pomace as an alternative to nitrates and nitrites, research is needed to determine the optimal amounts of the substitute that exhibit similar characteristics in processed meats. Future research should focus on improving pre-processing methods and ingredient incorporation to maximize the functional benefits of berry pomace. Furthermore, consumer perception and acceptance will be crucial for shaping product development strategies.

## Figures and Tables

**Table 1:** Composition and health-promoting properties of berry fruit pomace.

Type of Fruit	Main Bioactive Compounds in Fruit/Pomace	Health-Promoting Properties	References
Black currant (*Ribes nigrum*)	Anthocyanins: delphinidin-3-O-glucoside, delphinidin-3-O-rutinoside, cyanidin-3-O-rutinoside, derivatives of malvidin, petunidin, peonidin; flavonols: quercetin, kaempferol; phenolic acids: chlorogenic acid, caffeic acid-O-glucoside; vitamin C; dietary fiber; PUFA, phytosterols and tocopherols	Antioxidant and anti-inflammatory activity; support of the immune system and metabolism	[17,19,20,33]
Red currant (*Ribes rubrum*)	Anthocyanins: cyanidin-3-O-rutinoside, cyanidin-3-O-(2″-O-xylosyl) rutinoside; flavonoids: rutin, quercetin; phenolic acids: caffeic acid, 4-hydroxybenzoic-O-hexoside; vitamin C; dietary fiber;	Antioxidant and anti-inflammatory effects; support of the cardiovascular system and metabolism; reduction of the risk of osteoporosis, diabetes and cancer	[21,22,23,24,25]
Chokeberry (*Aronia melanocarpa*)	Anthocyanins, proanthocyanidins; flavonols; quercetin glycosides; hydroxycinnamic acids: chlorogenic and neochlorogenic acids; fiber: cellulose, hemicellulose, pectins	Antioxidant and anti-inflammatory activity; support of the circulatory system and metabolism	[16,26,27,28,29,32]
Raspberry (*Rubus idaeus*)	Anthocyanins: cyanidin-3-O-sophoroside, cyanidin-3-O-(2″-O-glucosyl)rutinoside, cyanidin-3-O-sambubioside, cyanidin-3-O-glucoside; ellagic acid derivatives; quercetin; dietary fiber	Antioxidant and anti-inflammatory activity; support of the digestive system	[17]
Blueberry (*Vaccinium* spp.)	Anthocyanins: delphinidin-, cyanidin-, petunidin-, malvidin-3-O-galactosides; 5-O-feruloylquinic acid; phenolic acids: gallic, chlorogenic, ellagic, ferulic acids; quercetin glycosides	Antioxidant activity; anti-inflammatory potential; support of the nervous system	[17,30]
Cranberry (*Vaccinium* spp.)	Anthocyanins; vitamin C; phenolic acids: p-coumaric, caffeic, ferulic acids; polyphenols	Antioxidant and antibacterial activity; support of the urinary system	[17,30,31]

**Table 2:** The use of various forms of berry fruit pomace in meat products.

Fruit	Form of Pomace Application	Meat Product	Observed Effects in the Product	References
Blueberry	Extract (2% m/m) to the meat mass	Pork meatloaf	Increased oxidative stability of the product during refrigerated storage	[91]
Blackberry	Extract (250 mL/1 kg) to the meat mass	Emulsified cooked burger patties Burgers patties	Reduced oxidation of muscle proteins (lower formation of protein carbonyl groups) and reduced lipid oxidation (lower TBARS) during heat treatment and storage	[92,93]
Black currant	Extract (0,1%; 0,3%; 0,5% (m/m) to the meat mass	Pork patties	Reduction in TBARS values and lower formation of protein carbonyl groups	[94]
Black chokeberry, blackberry, red currant, blueberry	Extract (60 g/kg) to the meat mass	Beef patties	Reduction of TBARS values and inhibition of mesophilic, psychrotrophic bacteria and *E. coli* growth	[21]
Raspberry	Supercritical CO_2_ extract (SCE) (0.5–1.0%/kg), ethanol extract (ETE) (0.3–1.0%/kg) to the meat mass	Beef burgers	Lower pH, inhibition of microorganisms (*Enterobacteriaceae*, *Pseudomonas* spp.), improved oxidative stability and shelf life	[95]
Cranberry	Extract (0.125, 0.25, 0.50, 0.75, 1.00, and 1.25% wt/vol)	Fermented dry sausages	Reduction of *Salmonella enterica*, inhibition of *Staphylococcus* spp., color stabilization, reduced hardness	[56]
Blueberry	Freeze-dried (1%, 2%/kg) to the meat mass	Pork patties	Reduced formation of volatile lipid oxidation products (hexanal, heptanal), improved oxidative stability and increased stability and cooking efficiency	[96]
Black chokeberry	Fresh pomace (pressed and ground pomace to a “paste” consistency) (0.5; 1.0; 2.0; 3.5; 5.0; 7.0% (m/m) to the meat mass	Pork hamburgers	Lower pH, decreased lightness (L*), increased redness (a*)	[97]
Black chokeberry	Fresh pomace (pressed and ground pomace to a “paste” consistency) (2.0%, 3.5%, 5.0%) to the meat mass	Pork burgers	Decreased L*, increased a*, increased hardness	[88]
Raspberry	Spray-dried powder (1.0%, 2.0%, 3.0%) to the meat mass	Vacuum-packed ground beef	Lower microbial counts, extended shelf life	[98]
Blueberry	Fermented berries (2%, 4%, 6%) to the meat mass	Emulsion-type sausage	Reduction in TBARS (<1 mg MDA/kg) and reduced protein oxidation (lower formation of protein carbonyl groups)	[47]

## Data Availability

No new data were created or analyzed in this study. Data sharing is not applicable to this article.

## References

[1] Kaur R., Sharma M. (2019). Cereal polysaccharides as sources of functional ingredient for reformulation of meat products: A review. J. Funct. Foods.

[2] Sindelar J.J., Milkowski A.L. (2012). Human safety controversies surrounding nitrate and nitrite in the diet. Nitric Oxide.

[3] De Mey E., De Maere H., Paelinck H., Fraeye I. (2017). Volatile N-nitrosamines in meat products: Potential precursors, influence of processing, and mitigation strategies. Crit. Rev. Food Sci. Nutr..

[4] Bedale W., Sindelar J.J., Milkowski A.L. (2016). Dietary nitrate and nitrite: Benefits, risks, and evolving perceptions. Meat Sci..

[5] Beriain M.J., Gómez I., Ibáñez F.C., Sarriés V., Ordóñez A.I. (2018). Improvement of the functional and healthy properties of meat products. Food Quality: Balancing Health and Disease.

[6] Teixeira A., Rodrigues S. (2021). Consumer perceptions towards healthier meat products. Curr. Opin. Food Sci..

[7] Asioli D., Aschemann-Witzel J., Caputo V., Vecchio R., Annunziata A., Næs T., Varela P. (2017). Making sense of the “clean label” trends: A review of consumer food choice behavior and discussion of industry implications. Food Res. Int..

[8] Mirabella N., Castellani V., Sala S. (2014). Current options for the valorization of food manufacturing waste: A review. J. Clean. Prod..

[9] Šojić B., Tomović V., Džinić N., Jokanović M., Ikonić P., Škaljac S., Pavlić B. (2019). Plant extracts as natural antioxidants in meat processing. Bulg. J. Agric. Sci..

[10] Piasecka I., Brzezińska R., Kalisz S., Wiktor A., Górska A. (2024). Recovery of antioxidants and oils from blackcurrant and redcurrant wastes by ultrasound-assisted extraction. Food Biosci..

[11] (2023). Eurostat. Agricultural Production—Crops. https://ec.europa.eu/eurostat/statistics-explained/.

[12] Mezzetti B. (2013). EUBerry: The Sustainable Improvement of European Berry Production, Quality, and Nutritional Value in a Changing Environment. Int. J. Fruit Sci..

[13] Jara-Palacios M.J., Santisteban A., Gordillo B., Hernanz D., Heredia F.J., Escudero-Gilete M.L. (2019). Comparative study of red berry pomaces (blueberry, red raspberry, red currant and blackberry) as source of antioxidants and pigments. Eur. Food Res. Technol..

[14] Kosmala M., Kołodziejczyk K., Markowski J., Mieszczakowska M., Ginies C., Renard C.M.G.C. (2010). Co-products of black-currant and apple juice production: Hydration properties and polysaccharide composition. LWT-Food Sci. Technol..

[15] Kapasakalidis P., Rastall R.A., Gordon M.H. (2009). Effect of a Cellulase Treatment on Extraction of Antioxidant Phenols from Black Currant (*Ribes nigrum* L.) Pomace. J. Agric. Food Chem..

[16] Nawirska-Olszańska A., Kwaśniewska M. (2005). Dietary fibre fractions from fruit processing waste. Food Chem..

[17] Borges G., Degeneve A., Mullen W., Crozier A. (2010). Identification of flavonoid and phenolic antioxidants in black currants, blueberries, raspberries, red currants, and cranberries. J. Agric. Food Chem..

[18] Van der Sman R. (2020). Impact of Processing Factors on Quality of Frozen Vegetables and Fruits. Food Eng. Rev..

[19] Jurgiel-Małecka G., Buchwał A. (2016). Characterization of blackcurrant fruit. Food Sci. Technol. Qual..

[20] Oczkowski M. (2021). Health-promoting effects of bioactive compounds in blackcurrant (*Ribes nigrum* L.) Berries. Ann. Natl. Inst. Hyg..

[21] Babaoğlu A.S., Unal K., Dilek N.M., Poçan H.B., Karakaya M. (2022). Antioxidant and antimicrobial effects of blackberry, black chokeberry, blueberry, and red currant pomace extracts on beef patties subject to refrigerated storage. Meat Sci..

[22] Michalska A., Wojdyło A., Lech K., Łysiak G.P., Figiel A. (2017). Effect of different drying techniques on physical properties, total polyphenols and antioxidant capacity of blackcurrant pomace powders. LWT.

[23] Iqbal A., Schulz P., Rizvi S.S.H. (2021). Valorization of bioactive compounds in fruit pomace from agro-fruit industries: Present Insights and future challenges. Food Biosci..

[24] Frum A., Georgescu C., Gigor F., Dobrea C., Tita O. (2017). Identification and Quantification of Phenolic Compounds from Red Currant (*Ribes rubrum* L.) and Raspberries (*Rubus idaeus* L.). Int. J. Pharmacol. Phytochem. Ethnomed..

[25] Zdunić G., Šavikin K., Pljevljakusic D., Djordjevic B. (2015). Black (*Ribes nigrum L*.) and Red Currant (*Ribes rubrum* L.) Cultivars. Nutritional Composition of Fruit Cultivars.

[26] Kulling S.E., Rawel H.M. (2008). Chokeberry (*Aronia melanocarpa*)—A Review on the Characteristic Components and Potential Health Effects. Planta Medica.

[27] Wu X., Gu L., Prior R.L., McKay S. (2004). Characterization of anthocyanins and proanthocyanidins in some cultivars of Ribes, Aronia, and Sambucus and their antioxidant capacity. J. Agric. Food Chem..

[28] Sójka M., Kołodziejczyk K., Milala J. (2013). Polyphenolic and basic chemical composition of black chokeberry industrial by-products. Ind. Crops Prod..

[29] Baranowski K., Baca E., Salamon A., Michałowska D., Meller D., Karaś M. (2009). Possibilities of retrieving and making a practical use of phenolic compounds from the waste products: Blackcurrant and chokeberry pomace and spent hops. Food Sci. Technol. Qual..

[30] Pedisić S., Zorić Z., Ćosić Z., Repajić M., Dragović-Uzelac V., Levaj B., Dobrinčić A., Balbino S., Elez Garofulić I. (2025). Valorization of Berry Fruit By-Products: Bioactive Compounds, Extraction, Health Benefits, Encapsulation and Food Applications. Foods.

[31] Struck S., Plaza M., Turner C., Rohm H. (2016). Berry pomace—A review of processing and chemical analysis of its polyphenols. Int. J. Food Sci. Technol..

[32] Reißner A.-M., Alhamimi S., Quiles A., Schmidt C., Struck S., Hernando I., Turner C., Rohm H. (2019). Composition and physicochemical properties of dried berry pomace. J. Sci. Food Agric..

[33] Górnaś P., Juhņeviča-Radenkova K., Radenkovs V., Mišina I., Pugajeva I., Soliven A., Segliņa D. (2016). The impact of different baking conditions on the stability of the extractable polyphenols in muffins enriched by strawberry, sour cherry, raspberry or black currant pomace. LWT.

[34] Lu J., Li M., Huang Y., Xie J., Shen M., Xie M. (2022). A comprehensive review of advanced glycosylation end products and N-Nitrosamines in thermally processed meat products. Food Control.

[35] Wang Y., Huang Y., Chai L. (2020). The effect of substituting nitrite with rose extract on the quality of semi-dried fermented sausage. Food Ferment. Ind..

[36] Ozaki M.M., Santos M., Ribeiro W.O., Ferreira N., Pollonio M. (2020). Radish powder and oregano essential oil as nitrite substitutes in fermented cooked sausages. Food Res. Int..

[37] Zhu Y., Guo L., Yang Q. (2020). Partial replacement of nitrite with a novel probiotic *Lactobacillus plantarum* on nitrate, color, biogenic amines and gel properties of Chinese fermented sausages. Food Res. Int..

[38] Leong Y.K., Chang J.-S. (2022). Valorization of fruit wastes for circular bioeconomy: Current advances, challenges, and opportunities. Bioresour. Technol..

[39] Chen J., Xia P. (2024). Health effects of synthetic additives and the substitution potential of plant-based additives. Food Res. Int..

[40] Inguglia E.S., Song Z., Kerry J.P., O’Sullivan M.G., Hamill R.M. (2023). Addressing Clean Label Trends in Commercial Meat Processing: Strategies, Challenges and Insights from Consumer Perspectives. Foods.

[41] Olanwanit W., Phahom T. (2025). Evaluation of Mao berry (*Antidesma bunius*) extracts as an alternative to sodium nitrite in emulsion sausages: Refrigerated storage quality and degradation kinetics. Int. J. Food Prop..

[42] Domínguez R., Pateiro M., Gagaoua M., Barba F.J., Zhang W., Lorenzo J.M. (2019). A Comprehensive Review on Lipid Oxidation in Meat and Meat Products. Antioxidants.

[43] Piechowiak T., Skóra B., Grzelak-Błaszczyk K., Sójka M. (2021). Extraction of Antioxidant Compounds from Blueberry Fruit Waste and Evaluation of Their In Vitro Biological Activity in Human Keratinocytes (HaCaT). Food Anal. Methods.

[44] Sarv V., Venskutonis P.R., Rätsep R., Aluvee A., Kazernavičiūtė R., Bhat R. (2021). Antioxidants Characterization of the Fruit, Juice, and Pomace of Sweet Rowanberry (*Sorbus aucuparia* L.) Cultivated in Estonia. Antioxidants.

[45] Zhang M.-Q., Zhang J., Zhang Y.-T., Sun J.-Y., Prieto M.A., Simal-Gandara J., Putnik P., Li N.-Y., Liu C. (2023). The link between the phenolic composition and the antioxidant activity in different small berries: A metabolomic approach. LWT.

[46] Zhou H., Zhuang X., Zhou C., Ding D., Li C., Bai Y., Zhou G. (2020). Effect of fermented blueberry on the oxidative stability and volatile molecule profiles of emulsion-type sausage during refrigerated storage. Asian-Australas. J. Anim. Sci..

[47] Skwarek P., Karwowska M. (2022). Fatty Acids Profile and Antioxidant Properties of Raw Fermented Sausages with the Addition of Tomato Pomace. Molecules.

[48] Skwarek P., Karwowska M. (2025). The Effect of Tomato Pomace on the Oxidative and Microbiological Stability of Raw Fermented Sausages with Reduced Nitrite Addition. Int. J. Food Sci..

[49] Koishybayeva A., Korzeniowska M. (2024). Utilization and Effect of Apple Pomace Powder on Quality Characteristics of Turkey Sausages. Foods.

[50] Yadav S., Malik A.K., Pathera A.K., Islam R.U., Sharma D.P. (2016). Development of Dietary Fibre Enriched Chicken Sausages by Incorporating Corn Bran, Dried Apple Pomace and Dried Tomato Pomace. Nutr. Food Sci..

[51] Aly A., Ali H.G.M., Eliwa N.E.R. (2019). Phytochemical screening, anthocyanins and antimicrobial activities in some berries fruits. J. Food Meas. Charact..

[52] Bartkiene E., Lele V., Sakiene V., Zavistanaviciute P., Ruzauskas M., Bernatoniene J., Jakstas V., Viskelis P., Zadeike D., Juodeikiene G. (2019). Improvement of the antimicrobial activity of lactic acid bacteria in combination with berries/fruits and dairy industry by-products. J. Sci. Food Agric..

[53] Das Q., Islam M.R., Marcone M.F., Warriner K., Diarra M.S. (2017). Potential of berry extracts to control foodborne pathogens. Food Control.

[54] Salaheen S., Nguyen C., Hewes D., Biswas D. (2014). Cheap extraction of antibacterial compounds of berry pomace and their mode of action against the pathogen *Campylobacter jejuni*. Food Control.

[55] Lau A.T.Y., Barbut S., Ross K., Diarra M.S., Balamurugan S. (2019). The effect of cranberry pomace ethanol extract on the growth of meat starter cultures, *Escherichia coli* O157:H7, *Salmonella enterica* serovar Enteritidis and *Listeria monocytogenes*. LWT.

[56] Perumal S., Mahmud R., Ismail S. (2017). Mechanism of Action of Isolated Caffeic Acid and Epicatechin 3-gallate from *Euphorbia hirta* against *Pseudomonas aeruginosa*. Pharmacogn. Mag..

[57] De Rossi L., Rocchetti G., Lucini L., Rebecchi A. (2025). Antimicrobial Potential of Polyphenols: Mechanisms of Action and Microbial Responses—A Narrative Review. Antioxidants.

[58] Martuscelli M., Esposito L., Mastrocola D. (2021). The Role of Coffee Silver Skin against Oxidative Phenomena in Newly Formulated Chicken Meat Burgers after Cooking. Foods.

[59] Bakhsh A., Cho C., Baritugo K.E., Kim B., Ullah Q., Rahmanc A., Park S. (2023). Characterization of plant-based meat alternatives blended with anthocyanins, chlorophyll, and various edible natural pigments. Int. J. Food Prop..

[60] Bakhsh A., Cho C., Baritugo K.A., Kim B., Ullah Q., Rahman A., Park S. (2023). Production and Analytical Aspects of Natural Pigments to Enhance Alternative Meat Product Color. Foods.

[61] Jurevičiūtė I., Keršienė M., Bašinskienė L., Leskauskaitė D., Jasutienė I. (2022). Characterization of Berry Pomace Powders as Dietary Fiber-Rich Food Ingredients with Functional Properties. Foods.

[62] Dinali M., Liyanage R., Silva M., Newman L., Adhikari B., Wijesekara I., Chandrapala J. (2024). Fibrous Structure in Plant-Based Meat: High-Moisture Extrusion Factors and Sensory Attributes in Production and Storage. Food Rev. Int..

[63] Lambeau K.V., McRorie J.W. (2017). Fiber supplements and clinically proven health benefits: How to recognize and recommend an effective fiber therapy. J. Am. Assoc. Nurse Pract..

[64] Dulf F., Andrei S., Bunea A., Socaciu C. (2012). Fatty acid and phytosterol contents of some Romanian wild and cultivated berry pomaces. Chem. Pap..

[65] De Filette M., Schatteman K., Geuens J. (2024). Characterization of Six Cold-Pressed Berry Seed Oils and Their Seed Meals. Appl. Sci..

[66] Sankowski L.V., Hennig L., Drusch S., Brückner-Gühmann M. (2023). Potential of redcurrant protein-enriched fractions as emulsifier in oil-water-emulsions. Future Foods.

[67] Čechovičienė I., Šlepetienė A., Gumbytė M., Paulauskienė A., Tarasevičienė Ž. (2023). Composition and Physicochemical Properties of Pomace of Various Cultivars of Blackberry (*Rubus fruticosus* L.). Horticulturae.

[68] Al Alawi A.M., Majoni S.W., Falhammar H. (2018). Magnesium and Human Health: Perspectives and Research Directions. Int. J. Endocrinol..

[69] Maqbool M.A., Aslam M., Akbar W., Iqbal Z. (2017). Biological importance of vitamins for human health: A review. J. Agric. Basic Sci..

[70] Rodriguez-Mateos A., Heiss C., Borges G., Crozier A. (2014). Berry (poly)phenols and cardiovascular health. J. Agric. Food Chem..

[71] Popović T., Šarić B., Martačić J.D., Arsić A., Jovanov P., Stokić E., Mišan A., Mandić A. (2022). Potential health benefits of blueberry and raspberry pomace as functional food ingredients: Dietetic intervention study on healthy women volunteers. Front. Nutr..

[72] Yang B., Kortesniemi M. (2015). Clinical Evidence on Potential Health Benefits of Berries. Curr. Opin. Food Sci..

[73] Nemli E., Ozkan G., Subasi B.G., Cavdar H., Lorenzo J.M., Zhao C., Capanoglu E. (2024). Interactions between proteins and phenolics: Effects of food processing on the content anddigestibility of phenolic compounds. J. Sci. Food Agric..

[74] Özdal T., Yalcinkaya İ.E., Toydemir G., Capanoglu E. (2018). Polyphenol–protein interactions and changes in functional properties and digestibility. Reference Module in Food Science.

[75] Zhang Q., Cheng Z., Wang Y., Fu L. (2021). Dietary protein–phenolic interactions: Characterization, biochemical-physiological consequences, and potential food applications. Crit. Rev. Food Sci. Nutr..

[76] Quan T.H., Benjakul S., Sae-Leaw T., Balange A.K., Maqsood S. (2019). Protein–polyphenol conjugates: Antioxidant properties, functionalities and their applications. Trends Food Sci. Technol..

[77] Song Z., Zhang H., Niu P., Lin S., Xue Y., Meng Y., Wang X.Y., Gong T., Guo Y.R. (2023). Fabrication of a novel antioxidant emulsifier through tuning the molecular interaction between soy protein isolates and young apple polyphenols. Food Chem..

[78] Lai X., Li Y., Lan W., Zhao L., Wang K., Hu Z., Liu X. (2025). Interactions between proteins and polyphenols in plant-based food: Insight of allergenicity and off-flavor reduction. Food Chem..

[79] Tornberg E. (2005). Effects of heat on meat proteins–Implications on structure and quality of meat products. Meat Sci..

[80] Ahmad S., Gokulakrishnan P., Giriprasad R., Yatoo M. (2015). Fruit-based natural antioxidants in meat and meat products: A review. Crit. Rev. Food Sci. Nutr..

[81] Comporti M., Signorini C., Buonocore G., Ciccoli L. (2002). Iron release, oxidative stress and erythrocyte ageing. Free Radic. Biol. Med..

[82] Lund M.N., Heinonen M., Baron C.P., Estévez M. (2011). Protein oxidation in muscle foods: A review. Mol. Nutr. Food Res..

[83] Xiong Y.L., Blanchard S.P., Ooizumi T., Ma Y. (2010). Hydroxyl radical and ferryl-generating systems promote gel network formation of myofibrillar protein. J. Food Sci..

[84] Li X., Liu C., Wang J., Li W., Lin B., Zhu W., Yi S., Li J. (2020). Tea polyphenols affect oxidative modification and solution stability of myofibrillar protein from grass carp (*Ctenopharyngodon idellus*). Food Biophys..

[85] Zhang K., Huang J., Wang D., Wan X., Wang Y. (2024). Covalent polyphenol–protein interactions in food processing: Formation mechanisms, quantification methods, bioactive effects, and applications. Front. Nutr..

[86] Cegiełka A., Piątkowska J., Chmiel M., Hać-Szymańczuk E., Kalisz S., Adamczak L. (2025). Changes in Quality Features of Pork Burgers Prepared with Chokeberry Pomace During Storage. Appl. Sci..

[87] Cegiełka A., Perchuć J., Pietrzak D., Chmiel M. (2024). An Attempt to use Black Chokeberry Pomace in the Production of Hamburgers. Food Biotechnol. Agric. Sci..

[88] Ignat I., Volf I., Popa V.I. (2011). A critical review of methods for characterisation of polyphenolic compounds in fruits and vegetables. Food Chem..

[89] Belwal T., Ezzat S.M., Rastrelli L., Bhatt I.D., Daglia M., Baldi A., Devkota H.P., Orhan I.E., Patra J.K., Das G. (2018). A Critical Analysis of Extraction Techniques Used for Botanicals: Trends, Priorities, Industrial Uses and Optimization Strategies. TrAC Trends Anal. Chem..

[90] Huang H.-W., Hsu C.-P., Yang B.B., Wang C.Y. (2013). Advances in the extraction of natural ingredients by high pressure extraction technology. Trends Food Sci. Technol..

[91] Ganhão R., Morcuende D., Estévez M. (2010). Protein oxidation in emulsified cooked burger patties with added fruit extracts: Influence on colour and texture deterioration during chill storage. Meat Sci..

[92] Ganhão R., Estévez M., Armenteros M., Morcuende D. (2013). Mediterranean Berries as Inhibitors of Lipid Oxidation in Porcine Burger Patties Subjected to Cooking and Chilled Storage. J. Integr. Agric..

[93] Jia N., Kong B., Liu Q., Diao X., Xia X. (2012). Antioxidant activity of black currant (*Ribes nigrum* L.) extract and its inhibitory effect on lipid and protein oxidation of pork patties during chilled storage. Meat Sci..

[94] Chang L.-W., Yen W.-J., Huang S.C., Duh P.-D. (2002). Antioxidant activity of sesame coat. Food Chem..

[95] Zalewska M., Otreszko-Arski A., Zalewski M. (2016). The influence of convective drying and freeze drying on the colour of selected fruits. Res. Teach. Equip..

[96] Onsaard E., Onsaard W. (2019). Microencapsulated vegetable oil powder. Microencapsulation–Processes, Technologies and Industrial Applications.

[97] Rezagholizadeh-Shirvan A., Soltani M., Shokri S., Radfar R., Arab M., Shamloo E. (2024). Bioactive compound encapsulation: Characteristics, applications in food systems, and implications for human health. Food Chem..

[98] Duan W., Guan G., Zhang H.-L., Wang F.-Z., Lu R., Li D.-M., Geng Y., Xu Z.-H. (2023). Improving flavor, bioactivity, and changing metabolic profiles of goji juice by selected lactic acid bacteria fermentation. Food Chem..

[99] Muzolf-Panek M., Waśkiewicz A., Kowalski R., Konieczny P. (2016). The Effect of Blueberries on the Oxidative Stability of Pork Meatloaf During Chilled Storage. J. Food Process. Preserv..

[100] Skrovánková D., Sumczynski D., Mlcek J., Jurikova T., Sochor J. (2015). Bioactive Compounds and Antioxidant Activity in Different Types of Berries. Int. J. Mol. Sci..

[101] Kryževičūtė N., Jaime I., Diez A.M., Rovira J., Venskutonis P.R. (2017). Effect of raspberry pomace extracts isolated by high pressure extraction on the quality and shelf-life of beef burgers. Int. J. Food Sci. Technol..

[102] Diez-Sánchez E., Quiles A., Hernando I. (2023). Use of Berry Pomace to Design Functional Foods. Food Rev. Int..

[103] Huda A.B., Parveen S., Rather S.A., Akhter R., Hassan M. (2014). Effect of Incorporation of Apple Pomace on the Physico-Chemical, Sensory and Textural Properties of Mutton Nuggets. Int. J. Adv. Res..

[104] Goztepe B., Kayacan S., Bozkurt F., Tomas M., Sagdic O., Karasu S. (2022). Drying kinetics, total bioactive compounds, antioxidant activity, phenolic profile, lycopene and β-carotene content and color quality of Rosehip dehydrated by different methods. LWT.

[105] Gricenko T., Zommere A., Kviesis J., Klavins L. (2026). Effect of Drying Temperature on Berry Press Residue Anthocyanin Stability and Profile. Front. Sustain. Food Syst..

[106] Peiretti P.G., Gai F., Zorzi M., Aigotti R., Medana C. (2020). The effect of blueberry pomace on the oxidative stability and cooking properties of pork patties during chilled storage. J. Food Process. Preserv..

[107] Anjum A., Sood M., Singh J., Bandral J.D., Gupta N., Bhat A. (2019). Microencapsulation and its applications in food industry. J. Pharmacogn. Phytochem..

[108] Marcillo-Parra V., Tupuna-Yerovi D.S., González Z., Ruales J. (2021). Encapsulation of bioactive compounds from fruit and vegetable by-products for food application—A review. Trends Food Sci. Technol..

[109] Santos S.S., Rodrigues L.M., Costa S.C., Madron G.S. (2017). Microcapsules of Blackberry Pomace (*Rubus Fruticosus*): Light and Temperature Stability. Chem. Eng. Trans..

[110] Aksu M.I., Turan E., Gülbandılar A., Tamtürk F. (2023). Utilization of spray-dried raspberry powder as a natural additive to improve oxidative stability, microbial quality and overcome the perception of discoloration in vacuum-packed ground beef during chilled storage. Meat Sci..

[111] Markkinen N., Laaksonen O., Nahku R., Kuldjärv R., Yang B. (2019). Impact of lactic acid fermentation on acids, sugars, and phenolic compounds in black chokeberry and sea buckthorn juices. Food Chem..

